# Serological diagnosis of pulmonary *Mycobacterium tuberculosis* infection by LIPS using a multiple antigen mixture

**DOI:** 10.1186/s12866-015-0545-y

**Published:** 2015-10-08

**Authors:** Peter D. Burbelo, Jason Keller, Jason Wagner, James S. Klimavicz, Ahmad Bayat, Craig S. Rhodes, Bassirou Diarra, Ploenchan Chetchotisakd, Yupin Suputtamongkol, Sasisopin Kiertiburanakul, Steven M. Holland, Sarah K. Browne, Sophia Siddiqui, Joseph A. Kovacs

**Affiliations:** Dental Clinical Research Core, National Institute of Dental and Craniofacial Research, National Institutes of Health, Bethesda, MD 20892 USA; Laboratory of Sensory Biology, National Institute of Dental and Craniofacial Research, National Institutes of Health, Bethesda, MD 20892 USA; Laboratory of Cell and Developmental Biology, National Institute of Dental and Craniofacial Research, National Institutes of Health, Bethesda, MD 20892 USA; Project SEREFO, University of Sciences, Techniques and Technologies of Bamako (USTTB), Bamako, Mali; Khon Kaen University, Khon Kaen, Thailand; Faculty of Medicine Siriraj Hospital, Mahidol University, Bangkok, Thailand; Faculty of Medicine Ramathibodi Hospital, Mahidol University, Bangkok, Thailand; Laboratory of Clinical Infectious Diseases, National Institute of Allergy and Infectious Diseases, National Institutes of Health, Bethesda, MD 20892 USA; Collaborative Clinical Research Branch, Division of Clinical Research, National Institute of Allergy and Infectious Diseases, National Institutes of Health, Bethesda, MD 20892 USA; Critical Care Medicine Department, NIH Clinical Center, National Institutes of Health, Bethesda, MD 20892 USA; Dental Clinical Research Core, NIDCR, 10 Center Drive, Building 10, Rm. 5 N102/106, Bethesda, MD 20892 USA

**Keywords:** Antibodies, Latent tuberculosis infection (LTBI), Luciferase immunoprecipitation systems (LIPS), *Mycobacterium tuberculosis*, Pulmonary TB, Serology

## Abstract

**Background:**

There is an urgent need for a simple and accurate test for the diagnosis of human Mycobacterium tuberculosis, the infectious agent causing tuberculosis (TB). Here we describe a serological test based on light emitting recombinant proteins for the diagnosis of pulmonary *Mycobacterium tuberculosis* infection.

**Methods:**

Luciferase Immunoprecipitation Systems (LIPS), a fluid-phase immunoassay, was used to examine antibody responses against a panel of 24 different M. tuberculosis proteins. Three different strategies were used for generating the constructs expressing the recombinant fusion M. tuberculosis proteins with luciferase: synthetic gene synthesis, Gateway recombination cloning, and custom PCR synthesis. A pilot cohort of African pulmonary TB patients was used for initial antibody screening and confirmatory studies with selected antigens were performed with a cohort from Thailand and healthy US blood donors. In addition to testing M. tuberculosis antigens separately, a mixture that tested seven antigens simultaneously was evaluated for diagnostic performance.

**Results:**

LIPS testing of a pilot set of serum samples from African pulmonary TB patients identified a potential subset of diagnostically useful *M. tuberculosis* antigens. Evaluation of a second independent cohort from Thailand validated highly significant antibody responses against seven antigens (PstS1, Rv0831c, FbpA, EspB, bfrB, HspX and ssb), which often showed robust antibody levels up to 50- to 1000-fold higher than local community controls. Marked heterogeneity of antibody responses was observed in the patients and the combined results demonstrated 73.5 % sensitivity and 100 % specificity for detection of pulmonary TB. A LIPS test simultaneously employing the seven *M. tuberculosis* antigen as a mixture matched the combined diagnostic performance of the separate tests, but showed an even higher diagnostic sensitivity (90 %) when a cut-off based on healthy US blood donors was used.

**Conclusion:**

A LIPS immunoassay employing multiple *M. tuberculosis* antigens shows promise for the rapid and quantitative serological detection of pulmonary TB.

**Electronic supplementary material:**

The online version of this article (doi:10.1186/s12866-015-0545-y) contains supplementary material, which is available to authorized users.

## Background

*Mycobacterium tuberculosis* (MTB) infects more than one-third of the global population and is one of the world’s leading causes of mortality, resulting in approximately 1.7 million deaths annually [1]. Despite T- and B-cell mediated immunity against MTB, approximately 30 % of individuals develop latent, asymptomatic infection (LTBI) following primary infection. If LTBI is left untreated, there is a 10 % life-time risk of developing active tuberculosis (TB), usually localized to the lung [2]. In HIV-infected patients, there is an even greater risk, ~10 % per year, with a higher incidence of disseminated infection [3]. Fortunately, prophylaxis for patients identified with latent MTB infection can greatly reduce the risk of subsequent active infection [4].

The diagnosis of active TB infection involves sputum smear microscopy, bacterial culture, and molecular methods [5]. XpertMTB, a nucleic acid amplification test, shows high sensitivity and specificity for the diagnosis of active pulmonary disease including for detecting rifamycin resistance [6]. In contrast to active TB, subjects with LTBI show no clinical or radiographic symptoms and molecular assays are not diagnostically useful [7]. Tuberculin skin testing is used for detecting latent infection, but it has poor specificity and requires patients to return for evaluation. Alternatively, interferon-γ release assays, which exploit T cell responses, are highly effective for detecting LTBI, yet these assays are technically complex and require several days to process [8].

Efforts to develop serological tests for identification of MTB infection have been ongoing for many years [9, 10]. However, no reported immunoassay using either single or multiple target antigens has shown high enough sensitivity (i.e. the ability to correctly identify those with the disease) and specificity (i.e. the ability to correctly identify those without the disease) to meet the requirements for clinical utility. Another current limitation of solid-phase immunoassays such as ELISA [11, 12], microbead immunoassay [13] and even whole proteome protein arrays [14], is that these assays are not robust and show relatively modest differences in antibody signals between MTB-infected patients and controls, making it difficult to identify infected patients. In addition, antibody-based testing is complicated by the marked heterogeneity in humoral responses of TB-infected patients requiring multiple antigens to achieve high sensitivity [11, 13].

Unlike solid phase immunoassay, fluid-phase immunoassays show the highest sensitivity and specificity for detecting antibodies because they employ native antigens and efficiently detect conformational epitopes [15]. One such fluid-phase immunoassay employing light-emitting antigens, luciferase immunoprecipitation systems (LIPS), has been used to profile antibodies against a variety of infectious agents including viruses, fungi, filaria, and bacteria [16]. LIPS utilizes a *Renilla* luciferase enzymatic reporter with linear detection in light units for a concentration range over 7 orders of magnitude. In this report, LIPS was used to screen antibody responses against potential MTB antigens, resulting in the identification of seven antigens for the diagnosis of pulmonary TB. The feasibility of using seven MTB antigens in a mixture for the facile and robust detection of active TB infection is also demonstrated.

## Methods

### Clinical participants

The studies were approved by Institutional Review Board of the National Institute of Allergy and Infectious Diseases, NIH and written consent was obtained for all subjects. Three different cohorts of controls and/or subjects with TB from different geographical locations were employed (Additional file 1: Table S1). All patients were culture positive for MTB and HIV negative. In a pilot study, a small set of serum samples (cohort 1) from TB patients with pulmonary disease (*n* = 14) from Mali, Africa were screened against multiple potential antigens to identify those with potential clinical utility. A second cohort of serum samples from Thailand (cohort 2; *n* = 78) that included healthy control subjects (*n* = 22), patients with pulmonary TB (*n* = 49), and patients with extrapulmonary TB (*n* = 7) was used for validation. All TB patients in cohort 2 were on anti-TB treatment (average treatment of 3 months and 17 on treatment for 1–6 months within inactive and one with active disease) at the time of obtaining blood samples. Since there is a high level of LTBI in Thailand, and the control subjects from cohort 2 were not evaluated for LTBI by TST or interferon-γ release assays, sera from 16 healthy US donors (cohort 3) were used as additional controls.

### Selection of antigen for generating *Renilla* luciferase MTB fusion proteins

Based on published studies describing the serological diagnosis of TB [11–14, 17], a panel of twenty four MTB proteins (ESAT-6, TB16.3, TB9.7, TB15.3, TB 9.4, MPT63, L7 ribosomal, PTRP, cfp10, EspB, FbpC, Ssb, Mpt70, PstS1, Mpt64, BfrB, Rv0831C, PPE42, TIG, FbpA, LprG, CysA2, HspX and Pks10) was chosen for LIPS testing.

### Synthetic genes for generating *Renilla* luciferase MTB fusion proteins

Initially, nine MTB targets were generated as synthetic, human codon-optimized genes (Blue Heron Biotechnology, Seattle, WA) (Table 1), essentially as described [16, 18]. These synthetic genes were used as templates in PCR with primer adapter sequences to amplify each protein’s coding region. Following restriction enzyme digestion of the corresponding PCR products, the DNA fragments were ligated either downstream or upstream of the restriction enzyme-digested pREN2 (for C-terminal antigen fusions) and pREN3S (for N-terminal antigen fusions) *Renilla* luciferase expression vectors [18]. Subsequently, plasmids containing the correct insert were grown up and used to prepare plasmid DNA using a Qiagen Midi kit. DNA sequencing was used to confirm the integrity of the nine different MTB constructs.

**Table 1:** LIPS testing of TB proteins using cohort 1 sera

Rv number	Protein Name	Seropositivity^a^ as a C-terminal Gateway fusion protein (pREN5-ATT)	Seropositivity^a^ as a C-terminal fusion protein (pREN2)	Seropositivity^a^ as a N-terminal fusion protein (pREN3S)
Rv3875*	ESAT-6		0/14	0/14
Rv2185c*	TB16.3		1/14	0/14
Rv3354*	TB9.7		0/14	0/14
Rv1636*	TB15.3		0/14	
Rv3208A*	TB9.4			ND
Rv1926c*	Mpt63			0/14
Rv0652*	L7			7/14
Rv0538*	PTRP		0/14	
Rv3874*	cfp10	0/14	1/14	1/14
Rv3881c	EspB	0/14	6/14	4/14
Rv0129c	FbpC	0/14	0/14	0/14
Rv0054	Ssb	0/14	0/14	1/14
Rv2875	Mpt70	0/14	0/14	0/14
Rv0934	PstS1	0/14	1/14	
Rv1980c	Mpt64	0/14	2/14	
Rv3841	BfrB	0/14	4/14	
Rv0831c	Hypoth.	0/14	4/16	
Rv2608	PPE42	0/14	0/14	
Rv2462c	TIG	ND		
Rv3804c	FbpA	0/14	4/14	
Rv1411c	LprG	ND		
Rv0815c	CysA2		0/14	0/14
Rv2031c	HspX		1/14	0/14
Rv1660	Pks10		0/14	

^a^For screening of MTB proteins with Cohort 1 patients (*n* = 14), shown is the number of seropositive samples detected with values greater than twice the buffer blanks

*ND* not determined due to poor LU activity

Rv numbers with an asterisk denotes MTB antigen DNA created from synthetic genes

### Generation of *Renilla* luciferase-antigen fusion constructs by Gateway recombination

Constructs for thirteen additional MTB proteins (Table 1) were generated from Gateway MTB clones obtained from BEI resources (Manassas, Virginia), which were initially created through NIAID’s Pathogen Functional Genomics Resource Center (managed and funded by the Division of Microbiology and Infectious Diseases, NIAID, NIH, DHHS and operated by the J. Craig Venter Institute). In order to use this novel reagent set, a Gateway compatible destination vector was constructed to generate C-terminal fusion proteins with *Renilla* luciferase. To build this acceptor vector, a mammalian expression plasmid, pcDNA-Myc-Ruc, was first generated for expressing an N-terminal Myc-tag in frame with *Renilla* luciferase. Testing of the pcDNA-Myc-Ruc vector with several previously described antigenic targets revealed that protein expression was equivalent to that seen with the standard pREN2 vector (data not shown). The pcDNA-Myc-Ruc plasmid was further modified to produce a Gateway vector (pREN5-ATT), containing ATT recombination sites flanking the CCB toxic gene for accepting in-frame genes from Gateway donor clones. Gateway recombination reactions for generating the MTB antigens were then performed essentially as described [19]. Briefly, miniprep DNA was obtained from preexisting Gateway MTB donor clones grown under kanamycin selection. Miniprep DNA (50 ng) was then mixed with the ampicillin-resistant pREN5-ATT Gateway vector (50 ng) and incubated with LR clonase II enzyme mix (Invitrogen). Following bacterial transformation and selection of clones with ampicillin and carbenicillin, correctly recombined plasmids were checked by restriction enzyme digest and then confirmed by DNA sequencing.

### Custom construct design for generating *Renilla* luciferase-MTB antigen fusions

A third set of fifteen *Renilla* luciferase MTB constructs, lacking Gateway recombination and extra linker sequences before and after the coding sequence of each gene, were generated by PCR with gene-specific primers (Table 1). For each *Renilla* luciferase-MTB antigen construct generated in the pREN2 vector [18], the signal sequences for the proteins were omitted and a stop codon was included at the end of the coding sequence. Since several of the MTB proteins are extracellular, additional N-terminal MTB-*Renilla* luciferase fusions were constructed with the pREN3S vector [20] fusion constructs were created in some cases. The primer adapter sequences used to clone each protein or protein fragment are available upon request.

### LIPS analysis

**Fig. 1:**
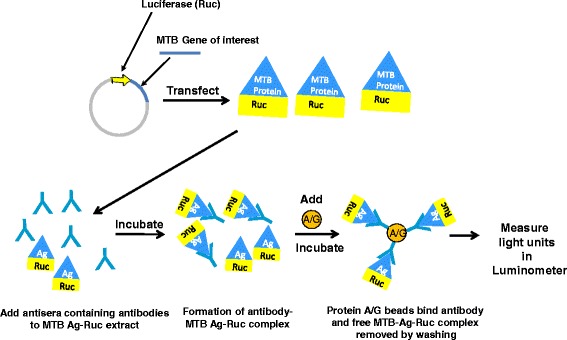
Schematic of the LIPS assay. DNA sequence of the MTB antigens of interest are genetically fused to the N- or C-terminus of *Renilla* luciferase (Ruc). These recombinant plasmids are then used to transfect Cos1 cells and cell lysate is harvested without purification. Aliquots of extract containing the Ruc-MTB antigen are then incubated with serum samples. The antibody complexes are then captured by protein A/G beads and the unbound luciferase tagged antigen is removed by extensive washing. The amount of specific antibody is determined by the amount of bound MTB-Ruc antigen present, which is determined by adding luciferase substrate and detection of luminescence

A schematic of the LIPS assay technology is shown in Fig. 1. LIPS assays were performed in a 96-well plate format at room temperature as described [21]. For these assays, transfected Cos1 cells were lysed with assay buffer A (20 mM Tris, pH 7.5, 150 mM NaCl, 5 mM MgCl_2_, 1 % Triton X-100) and centrifuged to obtain the crude lysates containing *Renilla* luciferase-MTB fusion proteins. Testing was performed using a master plate, which involved first diluting subject sera 1:10 in assay buffer A in a 96-well polypropylene microtiter plate. To initiate testing, 40 μl of buffer A, 10 μl of diluted human sera (1 μl equivalent), and *Renilla* luciferase-antigen Cos1 cell extract, diluted in buffer A, were added to each well of a polypropylene working plate and incubated for 1 h at room temperature. Next, 6 μl from a 30 % suspension of Ultralink protein A/G beads (Pierce Biotechnology, Rockford, IL) in PBS were added to the bottom of each well of a 96-well filter HTS plate (Millipore, Bedford, MA). The 100-μl antigen-antibody reaction mixture was transferred to this filter plate and incubated for 1 h at room temperature on a rotary shaker. The washing steps of the retained protein A/G beads were performed on plate washer with an integrated vacuum manifold (Tecan). After the final wash, light units (LU) were measured in a Berthold LB 960 Centro microplate luminometer (Berthold Technologies, Bad Wildbad, Germany) using coelenterazine substrate mix (Promega, Madison,WI). LU data were averaged from at least two separate experiments.

The method was modified slightly for the LIPS mixture based assay. In this case, each of the *Renilla* luciferase-MTB antigen extracts was harvested in lysis buffer without glycerol and used immediately upon collection to create a cocktail. Approximately 10 million LU from each of the seven MTB antigen lysates was combined in a single well with sera and then processed as above.

### Data analysis

GraphPad Prism 6 software (San Diego, CA) was used for statistical analysis. Antibody levels are presented as the geometric mean plus/minus 95 % confidence intervals. For the calculation of sensitivity and specificity, cut-off limits for each antigen were derived from the mean value plus three standard deviations of the Cohort 2 controls. Non-parametric Mann–Whitney *U* tests were used to compare the antibody levels among groups. Tests for differences between for the pulmonary TB patients and controls with the eight MTB antigens used a Bonferroni adjusted *P* value (*P* = 0.006) for significance. For heat map analysis, Cohort 2 controls were used as a reference group and the level of each antibody for each TB subject above the mean plus three standard deviations of the control values was calculated as a *Z* score value and then color-coded. For mixture testing, two different cut-off values were calculated based on the mean value plus three standard deviations (SD) of cohort 2 and cohort 3 control subjects.

## Results and discussion

### Using LIPS to study antibody responses against MTB proteins

As shown in Fig. 1, the LIPS technology employing *Renilla* luciferase-MTB antigen fusion proteins was used to examine antibody responses in TB patients. Based on the literature [11–14, 17], a panel of twenty four different MTB proteins was chosen for production as *Renilla* luciferase fusion proteins. Three different strategies, synthetic gene synthesis, Gateway recombination cloning, and custom PCR synthesis, were employed to generate the *Renilla* luciferase plasmids that produced the recombinant MTB antigen fusions. Using these MTB antigens, a potential LIPS test for the serological diagnosis of TB was developed based on the outlined strategy (see Additional file 2: Figure S2).

### LIPS testing of MTB antigens generated by synthetic genes

As described in the material and methods, nine of the twenty four constructs for expression of MTB proteins utilized synthetic gene synthesis. Initial screening of these nine antigens was performed with a pilot cohort of sera from 14 pulmonary TB patients out of Mali, Africa (cohort 1). Six proteins showed no significant seropositivity with the TB patient sera from cohort 1, but two antigens, cfp10 and Mpt63, showed potentially diagnostically useful immunoreactivity (Table 1). One additional protein, ribosomal L7 protein, reacted with TB patient sera, but subsequent testing of sera from healthy US donors (cohort 3) revealed high levels of seropositivity and this antigen was not studied further (data not shown).

### LIPS testing of MTB antigens generated by Gateway cloning

To test a larger number of potential antigens, an alternate strategy was utilized to produce recombinant proteins based on available Gateway clones encoding over three thousand different MTB proteins previously generated by the Pathogen Resource Center (Craig Venter Institute). However, testing of all thirteen additional MTB proteins, generated by Gateway recombination, failed to show immunoreactivity with any of the pulmonary TB patients from cohort 1 (Table 1). Notably, several antigens including EspB, FbpA and PstS1, previously shown to be serologically targets in multiple other studies [11, 13, 14], were not seropositive in LIPS using recombinant proteins produced by the Gateway *Renilla* luciferase vector.

### LIPS testing of MTB antigens generated by custom design

Since the Gateway constructs added additional amino acid sequences before the start methionine (TSLYKKVAH) and after the carboxyl-terminus stop codon (KLATFLYKVVTRACI) of each protein, we explored the possibly that these sequences might interfere with protein antigenicity. To determine the effect of removal of the Gateway sequences on antigenicity, thirteen of the proteins produced by the Gateway vector plus 3 additional targets were generated as custom MTB-*Renilla* luciferase constructs without the extra sequences (Table 1). Testing of the new MTB proteins absent the Gateway recombination sequences showed promising results, with eight of the thirteen proteins, including EspB, PstS1, FbpA, BfrB, Rv0831C, Mpt64, HspX and Ssb, binding high amounts of antibody in sera from a subset of patients (Table 1). EspB protein showed the highest seroprevalence of 50 % (7/14), while the seroprevalence of the other targets varied from 10 to 40 % (Table 1). These findings suggest that the extra Gateway peptides likely interfere with the proper folding of the *Renilla* luciferase MTB antigens needed to efficiently detect antibody responses.

### Robust detection of antibody responses against TB protein by LIPS

**Fig. 2:**
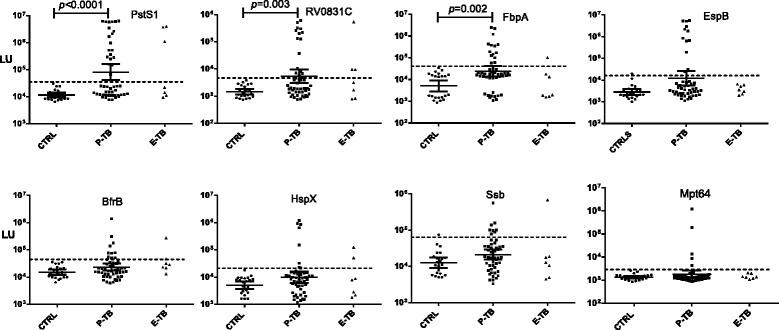
LIPS detection of antibody levels to the eight most informative MTB proteins. Antibodies against eight antigens including PstS1, FbpA, Rv0831C, EspB, Ssb, HspX, BfrB and Mpt64, were measured by LIPS. Each symbol represents individual samples from cohort 2 including 53 with pulmonary TB, 7 with disseminated TB and 22 controls. Each symbol represents individual subjects with antibody levels in light units (LU) plotted on the Y-axis using a log_10_ scale. The cut-off value for each antigen is shown by the dotted line and was based on the mean plus 3SD of the control group. Only statistically significant *p* values between the controls and the TB patients are shown and were calculated using the Mann Whitney *U* test with a Bonferroni adjusted *P* value = 0.006

To evaluate the potential diagnostic utility of the ten potential MTB antigens identified in the pilot study, an independent validation cohort from Thailand (cohort 2), consisting of serum samples from 49 patients with pulmonary TB, 7 with extrapulmonary TB, and 22 healthy controls, was used. As shown in Fig. 2, eight of the MTB antigens showed antibody levels up to 10–1000 times higher in individual TB patients than controls, especially in patients with pulmonary TB. Two antigens identified in the pilot cohort, TB-16.3 and cfp10, were not useful in cohort 2 and were not studied further. The raw values for these assay results are presented (see Additional file 3: Table S2). Using cut-off values derived from mean plus three standard deviations of the cohort 2 controls, PstS1, Rv0831c, FbpA, EspB, BfrB, HspX, Ssb and Mpt64 demonstrated sensitivity values of 41 %, 35 %, 29 %, 24 %, 22 %, 16 %, 16 %, and 10 %, respectively, for the detection of pulmonary TB. While EspB and Ssb showed 96.5 % specificity, the six other antigens showed 100 % specificity. Several of the patients with extrapulmonary TB also had significant antibody levels against 6 of the 8 antigens (Fig. 2). Additional testing of cohort 3 controls with a subset of the MTB antigens demonstrated values below those of cohort 2 controls (data not shown); these differences may be due to the higher incidence of LTBI in Thailand than in the United States. Lastly, many of these eight MTB proteins have important biological activities (Additional file 4: Table S3).

**Fig. 3:**
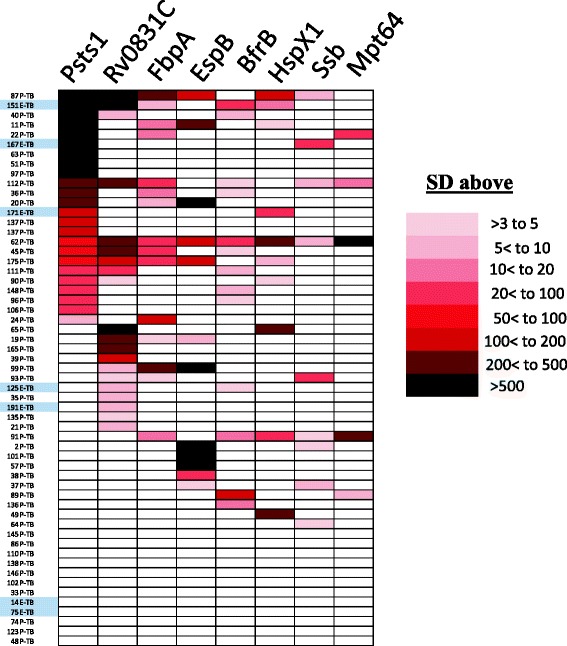
Heat map representation of patient antibody profiles against MTB antigens. Antibody responses of each TB-infected subject against eight MTB antigens was color-coded using the Z-score scale shown on the right representing the number of standard deviations above the mean of the controls for that antigen. Coloring in the heat map indicates that the relative antibody levels are at least greater than the mean of the controls plus three standard deviations. The pulmonary TB (P-TB) and extrapulmonary TB (E-TB; grey coded) patients are listed on the left and rank ordered based on immunoreactivity with the eight MTB antigens

To further understand each TB patient’s individual immunoreactivity profile with the eight antigens, a heatmap was used. Heatmap presentation showed that the spectra and antibody levels against the eight antigens varied markedly among the TB patients from Thailand (Fig. 3). While significant antibody levels to at least one of the eight antigens were observed in 76 % (37/49) of the pulmonary TB patients, 24 % showed no significant serological responses (Fig. 3). Of the seropositive patients, 30 % had antibody responses against only one antigen. The optimal diagnostic sensitivity using the minimum number of MTB antigens required only seven proteins without Mpt64. This is because several other antigens (e.g., PstS1, FbpA, and Bfrb) already showed overlapping seropositivity with the same TB patients detected with MPT64, yet these other antigens had much higher sensitivity (Fig. 3). These findings of heterogeneous B-cell responses among the TB patients are consistent with other studies [11, 13, 14, 17] highlighting the need to evaluate several MTB antigens to achieve high diagnostic sensitivity.

### A LIPS mixture test for TB diagnosis

**Fig. 4:**
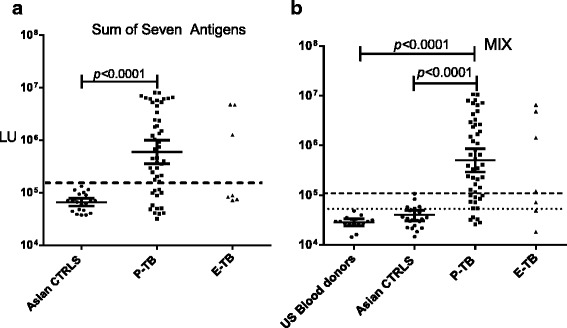
The sum of the seven individual tests or the LIPS mixture assay for TB diagnosis. **a** Antibodies in cohort 2 were evaluated by summation of the seven separate LIPS tests. The cut-off used (stippled line) was derived from the mean plus 3 standard deviations of controls from cohort 2. **b** A seven antigen LIPS mixture was used to evaluate antibodies in cohort 2 and an additional control set (cohort 3) of healthy US blood donors. The stippled line and dotted lines are the cut-offs derived from the mean plus 3 standard deviations of controls from cohort 2 and cohort 3, respectively

Since multiple antigens were needed to efficiently detect pulmonary TB, a diagnostic test based on the sum values of the seven separate LIPS antibody tests along with a cut-off value based on mean plus three standard deviations of the controls was employed. This approach of combining the LU values from the seven tests demonstrated 74 % sensitivity and 100 % specificity for diagnosis of pulmonary TB and closely tracked the overall diagnostic performance of the seven separate tests (Fig. 4a). Using this summed approach, three of the seven disseminated TB patients were also clearly identifiable as seropositive (Fig. 4a).

**Fig. 5:**
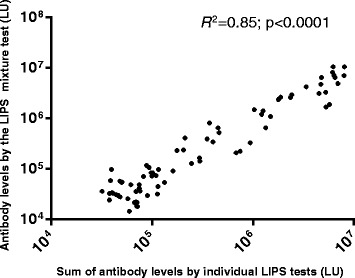
Comparison of the LIPS mixture test with the sum of seven individual tests for detecting antibodies for diagnosis of TB. Antibody levels from the LIPS mixture test (Y-axis) was plotted against the sum (X-axis) of the levels of the individual antibody levels against PstS1, Rv0831c, FbpA, EspB, BfrB, HspX and ssb. Each circle represents a TB patient or control subject. The correlation between the two tests showed an *R* ^2^ = 0.85

Based on our previous success with mixtures of antigens in the LIPS format for detecting and diagnosing autoimmune and infectious diseases [21–23], a mixture of the seven MTB antigens (PstS1, Rv0831c, FbpA, EspB, BfrB, HspX, and Ssb) was used to evaluate 1 μL serum samples from cohort 2, as well as from 16 additional US healthy blood donors (cohort 3 controls). The mixture test showed a striking difference between the control and TB patients: the geometric mean antibody level in the pulmonary TB group was 535,600 LU (95 % CI: 316,303–906,800 LU), significantly higher than the cohort 2 controls (35,480 LU; 95 % CI: 28,600–44,000 LU, *P* < 0.0001) and demonstrated 74 % sensitivity and 96 % specificity for pulmonary TB (Fig. 4b). The raw values for the mixture assay results are shown (Additional file 5: Table S4). The mixture test demonstrated a similar antibody profile to the summed individual tests (compare Fig. 4a and b) and there was also showed a strong correlation (*R*^2^ = 0.85; *p* < 0.0001) between the two results (Fig. 5). The combination of seven antigens reduced background binding to a level that was much lower than the summation of the individual tests, consistent with our previous mixture studies [16, 21]. The combined mixture format also detected three of the same disseminated TB patients as the summed approach, as well as one additional sample, which was likely due to lower background binding in the controls (Fig. 4b). Testing of cohort 3 US controls with the mixture showed even lower background values (Fig. 4b), resulting in 90 % sensitivity and 100 % specificity for detecting pulmonary TB, and identification of a fifth disseminated TB patient. Interestingly, seven of the 22 cohort 2 Asian controls were above this cut-off value (Fig. 4b), which is consistent with the possibility that these subjects had LTBI. The finding that 90 % of the pulmonary TB could be detected with the lower US healthy donor cut-off value is provocative, but requires future study.

Although studies using other immunoassays typically observe antibody titer differences of often less than ten-fold between healthy controls and TB patients [11–14, 17], LIPS showed much more robust detection of antibody responses. However, this required optimizing antigen design. Although the Gateway recombination system efficiently generated *Renilla* luciferase fusion proteins, remarkably no antigens produced using this approach detected patient antibody responses. In contrast, all of the diagnostically useful MTB antigens created by custom design produced much higher *Renilla* luciferase, likely reflecting their highly soluble nature and/or improved expression in mammalian cells. A major finding of our study was the high sensitivity (74–90 %) and specificity (96–100 %) achieved using the multi-antigen LIPS mixture. Compared to another study using MTB antigen-coated microbeads [13], LIPS demonstrated much more robust antibody signals to some of the same MTB antigens including PstS1, Rv0831C, FbpA and EspB. The LIPS mixture test also showed comparable sensitivity, but had a much higher specificity (i.e. 100 % for LIPS vs. 75–85 % for the multiplex beads). This LIPS mixture approach is highly attractive and avoids performing and interpreting the results from multiple immunoassays or engineering recombinant polyproteins [11]. Despite the robust detection of high levels of antibodies in some subjects, approximately 25 % of the pulmonary TB patients showed no significant antibody responses to any TB antigen. Screening of additional antigens or other improvements would be needed to determine if this represents total absence of TB-specific antibodies, or if responses do occur to other antigens.

One limitation of our study was that the geographic control group was not screened for LTBI. Since the prevalence of LTBI in Thailand is approximately 40 % [1], several of the control subjects were likely latently infected with TB. This is also consistent with our finding that approximately 30 % of the control subjects were seropositive if the cut-off based on US blood donors was used instead. Exploring whether LIPS can detect active and LTBI in a larger cohort is warranted.

## Conclusion

The LIPS, fluid-phase immunoassay detected robust antibody responses against MTB proteins, but required proper protein folding. Particularly attractive was the finding that the LIPS mixture format of seven antigens showed 74–90 % sensitivity and 96–100 % specificity. It is likely that the addition of new antigenic targets into the mixture format could further improve the performance. If optimized further, this LIPS mixture format might be useful for rapidly screening of patient samples in a 96-well format.

## Additional files

Additional file 1: Table S1. Characteristics of the cohorts used for LIPS testing. (PPT 79 kb) Additional file 2: Figure S1. Strategy used to develop a LIPS diagnostic test for TB. (PPT 72 kb) Additional file 3: Table S2. Raw LIPS data (LU) from separate tests for 8 MTB antigens with cohort 2. (XLS 18 kb) Additional file 4: Table S3. Biological activity of the eight informative MTB proteins. (PPT 86 kb) Additional file 5: Table S4. Raw LIPS data from the seven antigen mixture test with cohorts 2 and 3. (XLS 15 kb)

## Abbreviations

LIPS: Luciferase immunoprecipitation systems
MTB: *Mycobacterium tuberculosis*
TB: Active tuberculosis
LTBI: Latent tuberculosis infection
LU: Light units
Ruc: *Renilla* luciferase
SD: Standard deviations

## References

[1] Dye C, Scheele S, Dolin P, Pathania V, Raviglione MC (1999). Consensus statement. Global burden of tuberculosis: estimated incidence, prevalence, and mortality by country. WHO Global Surveillance and Monitoring Project. JAMA.

[2] Lin PL, Flynn JL (2010). Understanding latent tuberculosis: a moving target. J Immunol.

[3] Selwyn PA, Hartel D, Lewis VA, Schoenbaum EE, Vermund SH, Klein RS, Walker AT, Friedland GH (1989). A prospective study of the risk of tuberculosis among intravenous drug users with human immunodeficiency virus infection. N Engl J Med.

[4] Woldehanna S, Volmink J (2004). Treatment of latent tuberculosis infection in HIV infected persons. Cochrane Database Syst. Rev..

[5] Lalvani A (2007). Diagnosing tuberculosis infection in the 21st century: new tools to tackle an old enemy. Chest.

[6] Zeka AN, Tasbakan S, Cavusoglu C (2011). Evaluation of the GeneXpert MTB/RIF assay for rapid diagnosis of tuberculosis and detection of rifampin resistance in pulmonary and extrapulmonary specimens. J Clin Microbiol.

[7] Pai M, Kalantri S, Dheda K (2006). New tools and emerging technologies for the diagnosis of tuberculosis: part I. Latent tuberculosis. Expert Rev Mol Diagn.

[8] Herrera V, Perry S, Parsonnet J, Banaei N (2011). Clinical application and limitations of interferon-gamma release assays for the diagnosis of latent tuberculosis infection. Clin Infect Dis.

[9] Wallis RS, Pai M, Menzies D, Doherty TM, Walzl G, Perkins MD, Zumla A (2010). Biomarkers and diagnostics for tuberculosis: progress, needs, and translation into practice. Lancet.

[10] Pinto LM, Grenier J, Schumacher SG, Denkinger CM, Steingart KR, Pai M (2012). Immunodiagnosis of tuberculosis: state of the art. Med Princ Pract.

[11] Ireton GC, Greenwald R, Liang H, Esfandiari J, Lyashchenko KP, Reed SG (2010). Identification of Mycobacterium tuberculosis antigens of high serodiagnostic value. Clin Vaccine Immunol.

[12] Weldingh K, Rosenkrands I, Okkels LM, Doherty TM, Andersen P (2005). Assessing the serodiagnostic potential of 35 Mycobacterium tuberculosis proteins and identification of four novel serological antigens. J Clin Microbiol.

[13] Khan IH, Ravindran R, Krishnan VV, Awan IN, Rizvi SK, Saqib MA, Shahzad MI, Tahseen S, Ireton G, Goulding CW (2011). Plasma antibody profiles as diagnostic biomarkers for tuberculosis. Clin Vaccine Immunol.

[14] Kunnath-Velayudhan S, Salamon H, Wang HY, Davidow AL, Molina DM, Huynh VT, Cirillo DM, Michel G, Talbot EA, Perkins MD (2010). Dynamic antibody responses to the Mycobacterium tuberculosis proteome. Proc Natl Acad Sci U S A.

[15] Liu E, Eisenbarth GS (2007). Accepting clocks that tell time poorly: fluid-phase versus standard ELISA autoantibody assays. Clin Immunol.

[16] Burbelo PD, Lebovitz EE, Notkins AL (2014). Luciferase immunoprecipitation systems for measuring antibodies in autoimmune and infectious diseases. Transl Res.

[17] Khan IH, Ravindran R, Yee J, Ziman M, Lewinsohn DM, Gennaro ML, Flynn JL, Goulding CW, DeRiemer K, Lerche NW (2008). Profiling antibodies to Mycobacterium tuberculosis by multiplex microbead suspension arrays for serodiagnosis of tuberculosis. Clin Vaccine Immunol.

[18] Burbelo PD, Goldman R, Mattson TL (2005). A simplified immunoprecipitation method for quantitatively measuring antibody responses in clinical sera samples by using mammalian-produced Renilla luciferase-antigen fusion proteins. BMC Biotechnol.

[19] Sone T, Imamoto F (2012). Methods for constructing clones for protein expression in mammalian cells. Methods Mol Biol.

[20] Burbelo PD, Ching KH, Bush ER, Han BL, Iadarola MJ (2010). Antibody-profiling technologies for studying humoral responses to infectious agents. Expert Rev Vaccines.

[21] Burbelo PD, Leahy HP, Groot S, Bishop LR, Miley W, Iadarola MJ, Whitby D, Kovacs JA (2009). Four-antigen mixture containing v-cyclin for serological screening of human herpesvirus 8 infection. Clin Vaccine Immunol.

[22] Burbelo PD, Leahy HP, Iadarola MJ, Nutman TB (2009). A four-antigen mixture for rapid assessment of Onchocerca volvulus infection. PLoS Negl Trop Dis.

[23] Ching KH, Burbelo PD, Tipton C, Wei C, Petri M, Sanz I, Iadarola MJ (2012). Two major autoantibody clusters in systemic lupus erythematosus. PLoS One.

