# Supplementary material for: Serological diagnosis of pulmonary Mycobacterium tuberculosis infection by LIPS using a multiple antigen mixture
Source: BMC Microbiol. 2015 Oct 8;15:205. doi: 10.1186/s12866-015-0545-y (PMC4599810; doi:10.1186/s12866-015-0545-y)
Supplement: Additional file 1: Table S1. — Characteristics of the cohorts used for LIPS testing. (PPT 79 kb) [file 12866_2015_545_MOESM1_ESM.ppt]

## Slide 1
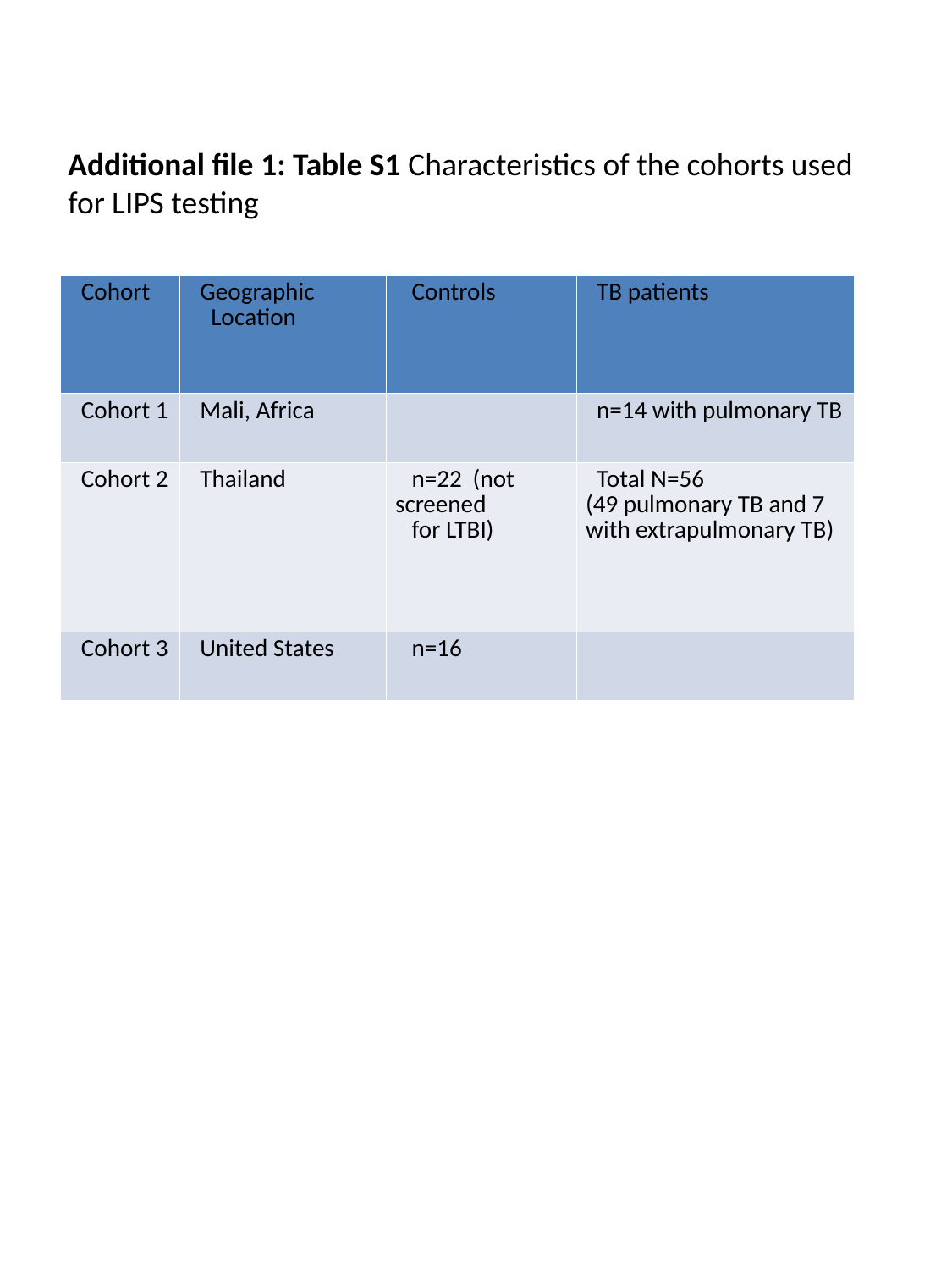

Additional file 1: Table S1 Characteristics of the cohorts used for LIPS testing
| Cohort | Geographic Location | Controls | TB patients |
| --- | --- | --- | --- |
| Cohort 1 | Mali, Africa | | n=14 with pulmonary TB |
| Cohort 2 | Thailand | n=22 (not screened for LTBI) | Total N=56 (49 pulmonary TB and 7 with extrapulmonary TB) |
| Cohort 3 | United States | n=16 | |
