# Supplementary material for: Serological diagnosis of pulmonary Mycobacterium tuberculosis infection by LIPS using a multiple antigen mixture
Source: BMC Microbiol. 2015 Oct 8;15:205. doi: 10.1186/s12866-015-0545-y (PMC4599810; doi:10.1186/s12866-015-0545-y)
Supplement: Additional file 2: Figure S1. — Strategy used to develop a LIPS diagnostic test for TB. (PPT 72 kb) [file 12866_2015_545_MOESM2_ESM.ppt]

## Slide 1
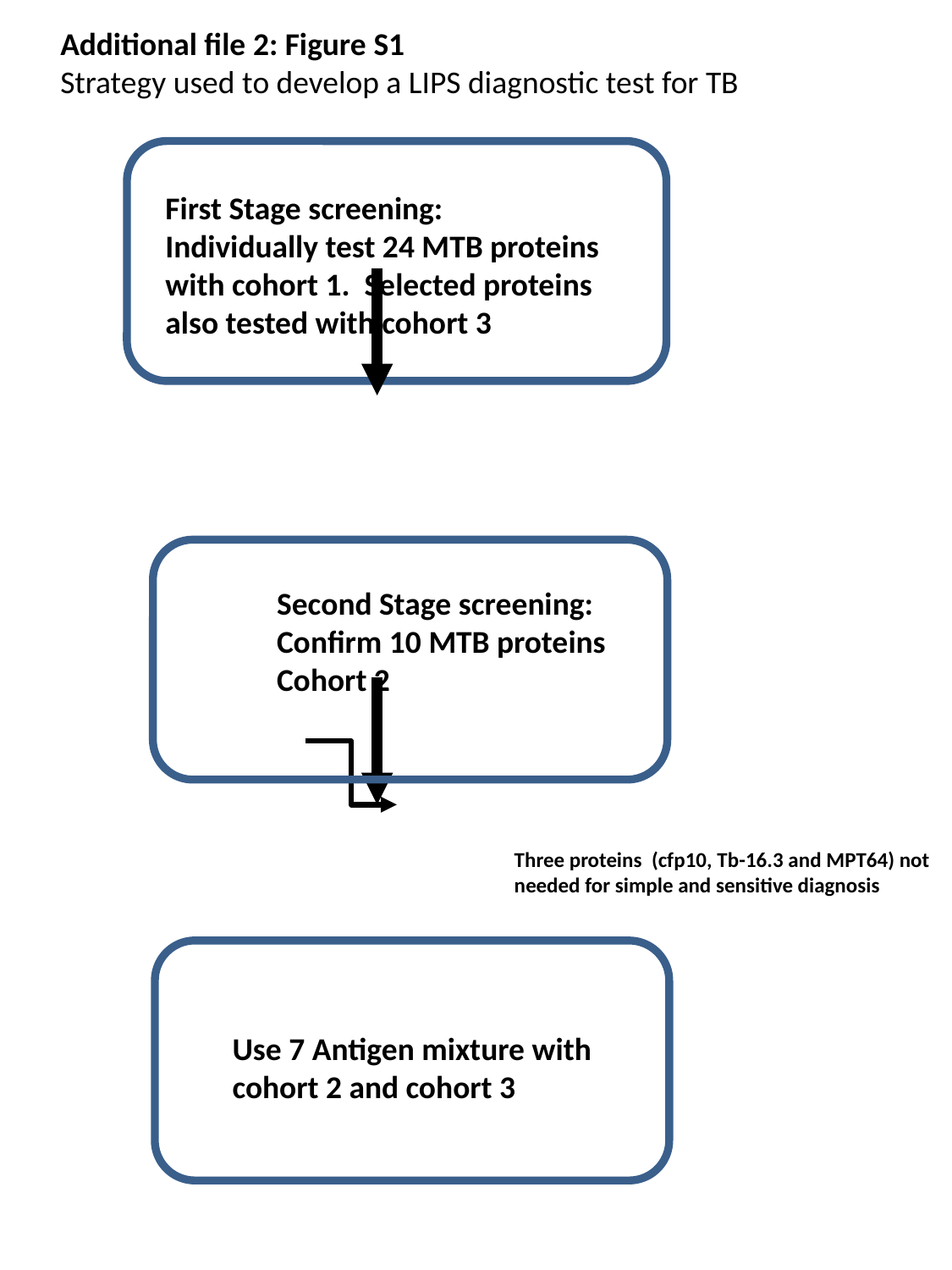

Additional file 2: Figure S1
Strategy used to develop a LIPS diagnostic test for TB
First Stage screening:
Individually test 24 MTB proteins with cohort 1. Selected proteins also tested with cohort 3
Second Stage screening:
Confirm 10 MTB proteins
Cohort 2
Three proteins (cfp10, Tb-16.3 and MPT64) not
needed for simple and sensitive diagnosis
Use 7 Antigen mixture with
cohort 2 and cohort 3
