# Supplementary material for: Serological diagnosis of pulmonary Mycobacterium tuberculosis infection by LIPS using a multiple antigen mixture
Source: BMC Microbiol. 2015 Oct 8;15:205. doi: 10.1186/s12866-015-0545-y (PMC4599810; doi:10.1186/s12866-015-0545-y)
Supplement: Additional file 4: Table S3. — Biological activity of the eight informative MTB proteins. (PPT 86 kb) [file 12866_2015_545_MOESM4_ESM.ppt]

## Slide 1
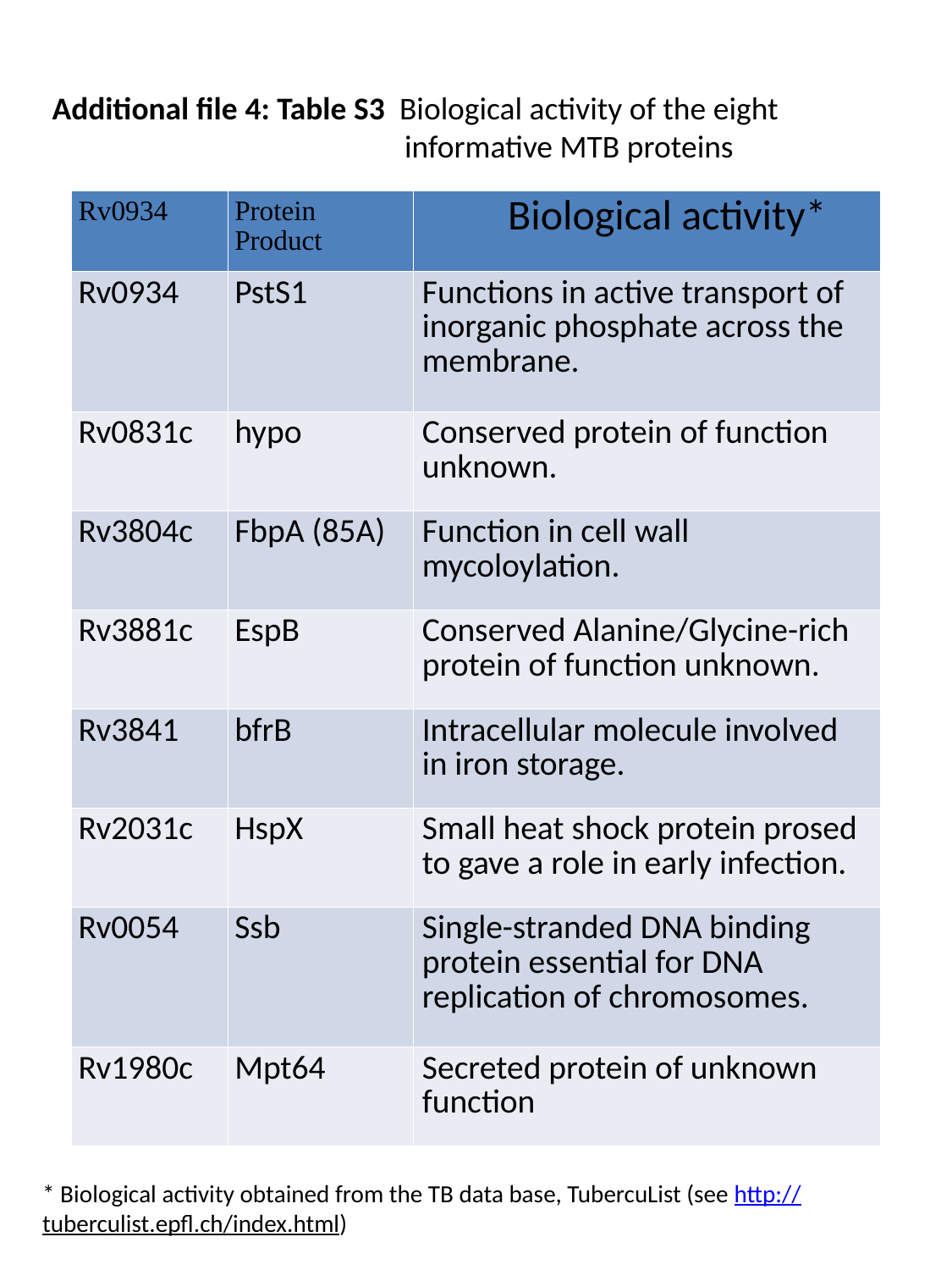

Additional file 4: Table S3 Biological activity of the eight
 informative MTB proteins
| Rv0934 | Protein Product | Biological activity\* |
| --- | --- | --- |
| Rv0934 | PstS1 | Functions in active transport of inorganic phosphate across the membrane. |
| Rv0831c | hypo | Conserved protein of function unknown. |
| Rv3804c | FbpA (85A) | Function in cell wall mycoloylation. |
| Rv3881c | EspB | Conserved Alanine/Glycine-rich protein of function unknown. |
| Rv3841 | bfrB | Intracellular molecule involved in iron storage. |
| Rv2031c | HspX | Small heat shock protein prosed to gave a role in early infection. |
| Rv0054 | Ssb | Single-stranded DNA binding protein essential for DNA replication of chromosomes. |
| Rv1980c | Mpt64 | Secreted protein of unknown function |
* Biological activity obtained from the TB data base, TubercuList (see http://tuberculist.epfl.ch/index.html)
